# Cord Blood Banking: Antenatal Care Provider's Roles and Responsibilities

**DOI:** 10.1155/2019/3598404

**Published:** 2019-03-07

**Authors:** Vishal Gupta, Lipisha Agarwal, Priya Ballal, Deeksha Pandey

**Affiliations:** ^1^Manipal Academy of Higher Education, Department of Obstetrics & Gynecology, Kasturba Medical College, Manipal 576104, India; ^2^Manipal Academy of Higher Education, Department of Obstetrics & Gynecology, Kasturba Medical College, Mangalore 575001, India

## Abstract

**Background:**

Umbilical Cord Blood (UCB) banking done either for private storage or for donation to public cord blood banks involves active participation of obstetricians. Counseling the expectant parents, providing them with unbiased and balanced information, and collecting the UCB with diligence confer a lot of social as well as moral responsibility upon obstetricians. This makes it even more important that the obstetricians in current practice stay well-informed and updated with UCB collection and its storage guidelines. The present study was conducted to assess the current status of obstetricians about UCB banking in terms of their awareness, attitude, and expectations from it.

**Materials and Methods:**

A cross-sectional study was conducted across three hospitals. A self-administered 22-item questionnaire was given to obstetricians to assess their awareness, attitude, and expectations about UCB banking. Finally, 154 completed questionnaires were analyzed using SPSS software (version 15.0). The awareness, attitude, and expectations were assessed and reported as primary endpoints and the self-rated knowledge levels, and sources of information were reported as secondary endpoints.

**Results:**

Overall, the awareness was poor, but the attitude was favorable for UCB banking amongst obstetricians. Around 74% felt that obstetricians must be well-informed about UCB banking-related counseling and collection protocols. However, 55% felt it to be an additional burden for the obstetrician, and 57% believed that financial compensation must be given to obstetricians involved with cord blood collection procedures. The majority remained unclear about their expectations from UCB banking. The self-rated knowledge was poor and very poor for 75% obstetricians. 89.6% derived their information from representatives of private cord blood companies.

**Conclusion:**

Although poor in awareness levels, obstetricians possessed a favorable attitude towards UCB banking. Continuing medical education needs to focus more on such current issues of public importance to keep professionals updated. This is one way to minimise percolation of wrong facts and figures by the industries with conflicting interest to the healthcare providers.

## 1. Introduction

Birth of a newborn is universally followed by the division of umbilical cord. The afterbirths are discarded as biological waste and channelized for appropriate disposal. After the successfully proven therapeutic utility of stem cells obtained from bone marrow and peripheral blood, the Umbilical Cord Blood (UCB) has emerged as a potentially promising source of stem cells which can be used for hematopoietic reconstitution [1, 2]. The practice and procedure of collection and storage of these UCB stem cells are called UCB banking.

The “triad” of this biobanking is formed by a donor mother, an obstetrician, and a UCB bank. The process of UCB collection is done by the obstetrician either upon request of the parents for private banking or for those who wish to donate their UCB to public banks for altruistic reasons. Currently, the American Academy of Pediatrics and the Society of Obstetrics and Gynecology of Canada clearly discourage the collection and storage of UCB for autologous banking due to its questionable significance for self-use in the future [3]. A detailed comparison of the two was given by Sullivan, stating that private banks publicise UCB banking in the disguise of “biological insurance” of an unborn child [4]. Patra and Sleeboom-Faulkner critically reviewed the UCB banking cycle in India and found an obvious disparity between prebanking persuasion and postbanking utilization [5].

For the purpose of UCB banking, the obstetricians are vital for counseling the pregnant mothers and after that for the actual process of the collection of cord blood itself. A recent review by Peberdy and colleagues established that expectant parents prefer to ask their obstetricians for more information on UCB banking and storage [6]. They also pointed out the lack of sufficient quality research on the assessment of obstetricians' awareness and attitude towards UCB banking. In addition to this, various studies done in different countries have pointed out that the obstetricians themselves are lacking in terms of awareness and understanding about UCB banking [7, 8], the situation being more serious in developing countries [9].

The awareness levels of obstetricians assume even more importance when it comes to public cord blood banking. As India also possesses a diverse ethnicity, maintaining a large public pool of donor units is very crucial to help maximum number of potential recipients.

Hence, the present study sought to examine the awareness, attitude, and expectations amongst obstetricians (an essential component of the triad) in a sample cohort from India which in fact is one of the largest potential repositories for public cord blood stem cell units.

## 2. Materials and Methods

The present study was conducted in three hospitals, affiliated to the same teaching institute of South India in a coastal district of Karnataka. All hospitals were nonpartner hospitals, unattached to any public cord blood banking establishments. Institutional Ethics Committee approval was obtained before commencing the study (IEC no: 623). Ethical practice was followed in accordance with the rules of the Declaration of Helsinki. A written informed consent was obtained from all participants, and they were encouraged to discuss their doubts and ask for any clarifications they had before participating in the study.

### 2.1. Study Population and Study Context

The study subjects consisted of all the obstetricians working in the Department of Obstetrics and Gynecology in three hospitals of coastal Karnataka in South India. The obstetricians were contacted personally for filling up the study questionnaire after obtaining a written and informed consent from them. The questionnaires were collected on the spot by the study investigator. The average time taken for filling the questionnaire was 11 minutes.

### 2.2. Study Variables Assessed

We aimed to assess the general awareness, attitude, and perceptions of obstetricians about UCB banking practices as the primary study variables.

A secondary analysis of self-rating of knowledge of obstetricians and their source of information about the same was also assessed.

### 2.3. Study Questionnaire

The study questionnaire was constructed to assess the awareness, attitude, and expectations of obstetricians. The questionnaire was designed after referring to various other studies done on healthcare practitioners and midwives. It was validated by five subject experts, one each from the departments of Hematology, Public Health, and Community Medicine and two from the Department of Obstetrics and Gynecology. A pilot study was first done on ten subjects, and questions were checked for any misunderstood options, unanswered questions, and unclear options. No major corrections were observed, and the final questionnaire with minor modifications was used for conducting the study.

Baseline demographics of the respondents like age, sex, number of years of experience in obstetrics, supervising residents or not, and the number of deliveries done per month were recorded. After that, the 22-item questionnaire was filled by the obstetricians. It consisted of five statements assessing the awareness of obstetricians about UCB banking [1–5] with responses recorded as correct or incorrect, ten statements assessing the attitude of obstetricians on UCB banking [6–15] with responses as Yes, No, or Do not know, and five statements on expectations of obstetricians from UCB banking (16-20) with responses coded as Yes, No, or Do not know. Finally, for secondary analysis, the subjects were asked to self-rate their knowledge about UCB banking on a Likert scale of 1-5 (1 being outstanding and five being very poor). The subjects were also asked to mention the source of their knowledge for UCB banking, and responses were coded and analyzed.

#### 2.3.1. Sample Size Calculation

A previous study done on a knowledge of UCB banking in North India reported awareness levels of doctors at 42% [9]. Therefore, with a relative precision of 20%, for a confidence level of 95%, a sample size of 133 was calculated. A total of 162 obstetricians were approached to fill up the questionnaire by study investigators. Out of 162, two refused to consent and fill out the questionnaire, and six returned incompletely filled questionnaires. Finally, 154 completed questionnaires were analyzed.

### 2.4. Statistical Analysis

The collected data was analyzed using SPSS software (version 15.0). The categorical variables (like gender, age, supervised resident teaching or not, postresidency work experience, and number of deliveries per month) were summarised and reported as counts and percentages.

The questions on awareness, attitude, and expectations and self-rated knowledge and sources of information were analyzed separately, and the responses were reported as counts and percentages.

## 3. Results

The present study on obstetricians looked into the awareness, attitude, and expectations from UCB banking in Southern India. A total of 162 obstetricians participated in the study, of which 154 completely filled and returned questionnaires were analyzed (Table 1). The median age was 37 years with 25 years being the youngest and 65 years being the oldest participant. More than one-third (70.1%) fell in the category of less than 40 years of age, representing the relatively younger participants in our study cohort. Majority of the study population was formed by female obstetricians (82.5%). As one of the three study hospitals had resident teaching programme in Obstetrics and Gynecology, there were 64.2% obstetricians who supervised them. About three-fourth of them had an experience of less than 15 years in obstetric practice (less than 5 years: 57.2%, 5-15 years: 20.8%), again representing a relatively younger mindset. Almost half of obstetricians (54.5%) dealt with less than 150 deliveries per month.

### 3.1. Awareness of UCB Banking

In this study, 57% obstetricians correctly knew the meaning of UCB banking. Only about one-third declared that they understood the concept of public and private UCB banking correctly (42.2% and 31.1%, respectively). A majority of respondents (69%) were unclear about its potential uses (Table 2).

### 3.2. Attitude on UCB Banking

The overall attitude on UCB banking was found to be favorable in this study. More than half felt that UCB banking must be recommended to all expectant parents (59%), that all antenatal care providers must be educated about utility and collection procedure of UCB banking (74%), and that doctors should counsel the expectant parents themselves and not rely on the representatives of private companies for information and counseling (52%). However, 48% of respondents did not know if India has its own public UCB bank. In addition to this, 84% declared that they were not aware of the international guidelines on UCB collection and banking and have not received any training directed towards it. When enquired about perceived difficulties associated with UCB banking, lack of financial compensation (57%) to the obstetrician is an important barrier to UCB banking. However, only 8% felt that it could seriously interfere with the labor and delivery process whereas 48% were not sure about it (Table 3).

### 3.3. Expectation from UCB Banking

More than half (51%) did not know if UCB can be used without complete HLA match and whether it can be stored for up to 20 years for future use. When asked about its definitive role in the treatment of cancer, 56% were sure about it. However, 54% and 49% were again unsure of its potential usage in the management of chronic illnesses like diabetes and hypertension and in regenerative medicine, respectively (Table 4).

**Figure 1:**
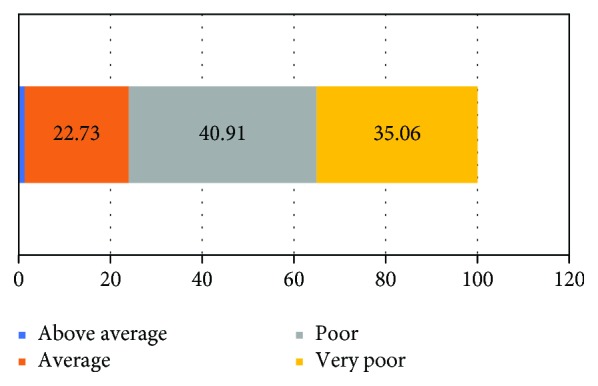
Self-rated knowledge of obstetricians of UCB banking.

About 75% of respondents self-rated their knowledge of UCB banking as either poor or very poor (Figure 1). When enquired about the sources of information about UCB banking, 89.6% derived it from the representatives of private cord blood banking companies (Table 5).

## 4. Discussion

The present study explored awareness, attitude, and expectations of the primary antenatal care providers, i.e., the obstetricians, about UCB banking. The general awareness levels were found to be poor amongst obstetricians with an overall positive attitude towards UCB banking. However, the obstetricians felt uncertain about their expectations from UCB banking. About 75% of obstetricians estimated their knowledge on UCB banking as poor and very poor, and majority derived the information from the representatives of private UCB banking companies.

In this study, though more than half (57%) understood exactly the correct meaning of UCB banking, two-third remained unclear about its potential uses. Similar findings were reported from North India in a survey done on both doctors and general public. They concluded that the general public suffered from total lack of knowledge on UCB banking and the doctors need to be sufficiently educated about the utility and potential of UCB banking [9]. Contrary to that, Walker and colleagues from the USA reported more than 80% knowledge levels amongst obstetricians in their survey on awareness and acceptance of public UCB banking [10]. This disparity in findings between developed and developing countries could be due to active involvement of the government and clear guidelines on UCB collection and banking by the authorities in the western world.

The obstetricians possessed an appreciative attitude on UCB banking in this study. Three-fourth (74%) opined that obstetricians must be educated and well-informed with the protocol of UCB collection and they themselves must provide authentic and unbiased information to the expectant parents. These findings were encouraging as the expectant parents preferred to ask their antenatal care providers for detailed information on UCB donation and banking. Herlihy and Delpapa also concluded that obstetricians should have a primary role in imparting this information [11]. Pandey and colleagues concluded that expectant mothers in their survey had very limited knowledge and undue expectations from UCB banking and their antenatal care providers should inform them about the pros and cons of the same [12]. A most recent study from Saudi Arabia suggested that women received the majority of information on UCB banking through social media and only 10% was through their obstetricians [13]. In addition to this, certain obstetric conditions require delayed cord clamping which must be discussed with the expectant parents. This is particularly important for parents seeking private storage of UCB as the delayed clamping of umbilical cord will result in much smaller and less useful cord blood units.

However, the finding that 48% were not aware if India has its own public UCB bank was an unimpressive one. This was also in contrast to the fact that Viswanathan et al. in 2009 declared that functioning of a public UCB bank needs cooperation and huge assistance from antenatal care providers in their summary of 7-year experience of a public UCB bank established by Reliance in India [14]. This focuses on the fact that efforts must go in creating awareness regarding public banks and organising sessions to acquaint the obstetricians with their functioning. Additionally, educational interventions imparting directed training towards UCB collection and banking are crucial for sustaining the knowledge levels. Similarly, in their study on maternity nurses in Egypt, Mohammed and EL Sayed reported a striking improvement from poor knowledge levels (88.7%) to good knowledge levels (90.7%) before and after an educational intervention [15].

The obstetricians in our study were found to be divided in their opinion on perceived barriers to UCB banking such as the collection procedure to be an extra burden for the obstetrician. These findings were almost similar to the survey done in Japan on obstetricians and midwives that UCB collection process is not an extra burden to obstetricians (68.4%), and it does not involve additional risks to the mother or newborn (84.2%) as the process is abandoned immediately if any complications are anticipated at the time of labor or delivery [16]. Similarly, many public UCB establishments have their staff to collect cord blood via ex vivo approach. This actually reduces the involvement of obstetricians for UCB collection. Lack of financial compensation to the obstetricians was also highlighted as a probable hurdle to UCB collection by Walker et al. [10]. The genuine expectations from UCB banking again divided the obstetricians with half of them unbelieving in their potential usage and their definitive role in the treatment of cancer. These findings reflect that as many as half of the respondents were ignorant that UCB stem cells have the advantage of being acceptable for transplantation even with 4/6 HLA match [14]. However, it holds true that UCB banking does come with stringent regulatory environment with additional burden of labelling and documentation which adds to the already existing complexity in an obstetrician's work atmosphere.

About 75% of respondents self-rated knowledge of UCB banking as either poor or very poor. This finding was found to be similar to a Croatian study on maternity staff where 62% declared that they need more information on UCB banking [17]. When enquired about the sources of information about UCB banking, 89.6% derived it from the representatives of private cord blood banking companies. Hatzistilli et al. found brochures from private cord blood companies to be the chief sources of information for midwives in Greece [18].

However, this study could not assess much about the concept of public UCB banking in detail. Firstly, the obstetricians themselves were either uncertain or ignorant about donating UCB to public banks. Secondly, India itself lacks at present a well-coordinated system for public UCB banking. Thirdly, obstetricians' knowledge of eligibility criteria for donors could not be assessed in the present study which is particularly important for the public UCB banking.

The ethnicity-specific nature of UCB transplantation makes the role of individually functioning public banks in different countries indispensable. However, the demands of cord blood collection procedure, strict temperature control requirements, logistics involved in its transportation, and then a high wastage rate of cord blood units make it mandatory for public banks to receive stable financial assistance from trusts and the central government. McKenna and Sheth highlighted that with India's booming birth rate, a public-private partnership model is needed for sustaining public UCB banks [19].

Within India, Relicord India (Mumbai), Jeevan Bank (Chennai), School of Tropical Medicine (Kolkata), and StemCyte (Ahmedabad) are offering public UCB banking services. Collectively, they have more than 5000 CBUs. Of these, only Jeevan Bank has listed its CBUs on the World Marrow Donor Association (WMDA) list. Unfortunately, Jeevan Bank stopped receiving further CBUs (as on 28 Feb 2017) for processing and storage because of serious financial constraints.

We suggest workshops and seminars need to be conducted as educational initiatives to promote awareness on UCB banking and clarify the benefits of public and private UCB banking. All residents in obstetrics and gynecology must receive directed training towards counseling and collection procedures as per the guidelines laid down by the Stem Cell Therapeutics and Research Act to maintain the international standards. This will also avoid wastage of collected CBUs at the time of processing and storage. The process of continuous educational efforts on UCB banking will ensure the optimal collection, storage, and usage of these stem cells in the future recipients.

## 5. Conclusion

The present study highlighted that obstetricians in a developing country like India did believe in the potential utility of UCB banking, but they are still not very clear about the donation of UCB to public banks. They do realize their primary role in counseling the expectant parents for UCB donation as well as their role in the collection process itself. But the poor knowledge levels mismatched against their own genuine belief that favors UCB banking.

## Figures and Tables

**Table 1:** Demographic background of obstetricians.

Demographic variable	*n* (%)
Age (in years)	
≤40 years	108 (70.1%)
>40 years	46 (29.9%)
Sex	
Male	27 (17.5%)
Female	127 (82.5%)
Supervise resident teaching	
Yes	99 (64.2%)
No	55 (35.8%)
Experience (in years, postresidency)	
<5 years	88 (57.2%)
5-15 years	32 (20.8%)
16-25 years	25 (16.2%)
>25 years	9 (5.8%)
Number of deliveries per month	
≤150	84 (54.5%)
>150	70 (45.5%)

**Table 2:** Awareness levels of obstetricians.

S.No.	Statement	Correct response *n* (%)	Incorrect response *n* (%)
(1)	UCB is a collection of blood from the placental side of the umbilical cord.	88 (57%)	66 (43%)
(2)	Public cord blood banking is where anyone can donate free of cost and anyone in need can take at some cost.	65 (42%)	89 (58%)
(3)	Private cord blood banking is where anyone can store at some cost and only family can use free of cost later.	48 (31%)	106 (69%)
(4)	The family history of metabolic & blood disorders should bank cord blood stem cells for the sake of their own family.	81 (53%)	73 (47%)
(5)	If 2500 have stored cord blood stem cells, one might need for self/family use.	62 (40%)	92 (60%)

**Table 3:** Attitude of obstetricians.

S.No.	Statement	Response *n* (%)		
(6)	UCB banking must always be recommended to all expectant parents.	Yes 91 (59%)	No 19 (12%)	Do not know 44 (28%)
(7)	All antenatal care providers must be educated about the utility and collection protocol for UCB banking.	Yes 114 (74%)	No 2 (38%)	Do not know 38 (25%)
(8)	All antenatal care providers must counsel their patients about UCB themselves and should not rely on representatives of private companies.	Yes 80 (52%)	No 25 (16%)	Do not know 49 (32%)
(9)	The collection procedure of UCB banking can seriously interfere in the birth of a newborn.	Yes 12 (8%)	No 68 (44%)	Do not know 74 (48%)
(10)	India has its own public UCB bank.	Yes 45 (29%)	No 35 (22%)	Do not know 74 (48%)
(11)	Are you familiar with the international guidelines/protocol for UCB collection and banking?	Yes 17 (11%)	No 129 (84%)	Do not know 8 (5%)
(12)	In the last five years, have you received any directed knowledge or training towards UCB?	Yes 25 (16%)	No 129 (84%)	Do not know
(13)	For a child born into your family, you will prefer UCB in a private bank.	Yes 51 (33%)	No 19 (12%)	Do not know 84 (55%)
(14)	UCBT is an extra burden for an obstetrician during the labor and delivery process.	Yes 85 (55%)	No 10 (7%)	Do not know 59 (38%)
(15)	Lack of financial compensation for obstetrician is an important barrier to UCB collection & banking.	Yes 88 (57%)	No 5 (3%)	Do not know 61 (40%)

**Table 4:** Expectations of obstetricians.

S.No.	Statement	Response *n* (%)		
(16)	UCB can be used without complete HLA match.	Yes 46 (30%)	No 29 (19%)	Do not know 79 (51%)
(17)	UCB can be stored up to 20 years for future usage.	Yes 63 (41%)	No 13 (8%)	Do not know 78 (51%)
(18)	UCB can be used for treating cancer.	Yes 86 (56%)	No 6 (4%)	Do not know 62 (40%)
(19)	UCB is useful in the management of chronic diseases like diabetes & hypertension.	Yes 17 (11%)	No 54 (35%)	Do not know 83 (54%)
(20)	UCB is useful in future research for the development of regenerative medicine techniques.	Yes 74 (48%)	No 4 (3%)	Do not know 76 (49%)

**Table 5:** Sources of information on UCB banking.

Sources	Yes *n* (%)	No *n* (%)
Magazines & books	17 (11.1%)	137 (88.9%)
Social media (Internet & mobile phones)	48 (31.2%)	106 (68.8%)
Seminars & conferences	54 (35.1%)	100 (64.9%)
Representatives from private cord blood bank companies	138 **(89.6%)**	16 (10.4%)

## Data Availability

The dataset used to support the findings of this study are included within the supplementary information file (S1).

## Supplementary Materials

Supplementary Materials . Supporting Information S1 Table: we confirm that the dataset that we have uploaded as S1 Table constitutes the underlying findings of our paper.
